# Prevalence of Multiplicity and Appropriate Adjustments Among Cardiovascular Randomized Clinical Trials Published in Major Medical Journals

**DOI:** 10.1001/jamanetworkopen.2020.3082

**Published:** 2020-04-17

**Authors:** Muhammad Shahzeb Khan, Maaz Shah Khan, Zunaira Navid Ansari, Tariq Jamal Siddiqi, Safi U. Khan, Irbaz Bin Riaz, Zain Ul Abideen Asad, John Mandrola, James Wason, Haider J. Warraich, Gregg W. Stone, Deepak L. Bhatt, Samir R. Kapadia, Ankur Kalra

**Affiliations:** 1 Department of Medicine, John H. Stroger, Jr. Hospital of Cook County, Chicago, Illinois; 2 Department of Medicine, Dow University of Health Sciences, Karachi, Pakistan; 3 Department of Medicine, Guthrie Robert Packer Hospital, Sayre, Pennsylvania; 4 Department of Medicine, Mayo Clinic, Rochester, Minnesota; 5 Division of Cardiovascular Medicine, Department of Internal Medicine, University of Oklahoma Health Sciences Center, Oklahoma City; 6 Division of Cardiovascular Medicine, Baptist Health Louisville, Louisville, Kentucky; 7 Medical Research Council Biostatistics Unit, Department of Biostatistics, University of Cambridge, Cambridge, United Kingdom; 8 Population Health Sciences Institute, Department of Biostatistics, Newcastle University, Newcastle upon Tyne, United Kingdom; 9 Cardiovascular Division, Department of Medicine, Brigham and Women’s Hospital, Harvard Medical School, Boston, Massachusetts; 10 Cardiology Section, Department of Medicine, Veterans Administration Boston Healthcare System, Boston, Massachusetts; 11 Cardiovascular Research Foundation, Department of Cardiovascular Medicine, Columbia University Medical Center, New York, New York; 12 Heart & Vascular Center, Brigham and Women’s Hospital, Harvard Medical School, Boston, Massachusetts; 13 Heart, Vascular & Thoracic Institute, Tomsich Family Department of Cardiovascular Medicine, Cleveland Clinic, Cleveland, Ohio

## Abstract

**Importance:**

Multiple analyses in a clinical trial can increase the probability of inaccurately concluding that there is a statistically significant treatment effect. However, to date, it is unknown how many randomized clinical trials (RCTs) perform adjustments for multiple comparisons, the lack of which could lead to erroneous findings.

**Objectives:**

To assess the prevalence of multiplicity and whether appropriate multiplicity adjustments were performed among cardiovascular RCTs published in 6 medical journals with a high impact factor.

**Design, Setting, and Participants:**

In this cross-sectional study, cardiovascular RCTs were selected from all over the world, characterized as North America, Western Europe, multiregional, and rest of the world. Data were collected from past issues of 3 cardiovascular journals (*Circulation*, *European Heart Journal*, and *Journal of the American College of Cardiology*) and 3 general medicine journals (*JAMA*, *The Lancet*, and *The New England Journal of Medicine*) with high impact factors published between August 1, 2015, and July 31, 2018. Supplements and trial protocols of each of the included RCTs were also searched for multiplicity. Data were analyzed December 20 to 27, 2018.

**Exposures:**

Data from the selected RCTs were extracted and verified independently by 2 researchers using a structured data instrument. In case of disagreement, a third reviewer helped to achieve consensus. An RCT was considered to have multiple treatment groups if it had more than 2 arms; multiple outcomes were defined as having more than 1 primary outcome, and multiple analyses were defined as analysis of the same outcome variable in multiple ways. Multiplicity was examined only for the analysis of the primary end point.

**Main Outcomes and Measures:**

Outcomes of interest were percentages of primary analyses that performed multiplicity adjustment of primary end points.

**Results:**

Of 511 cardiovascular RCTs included in this analysis, 300 (58.7%) had some form of multiplicity; of these 300, only 85 (28.3%) adjusted for multiplicity. Intervention type and funding source had no statistically significant association with the reporting of multiplicity risk adjustment. Trials that assessed mortality vs nonmortality outcomes were more likely to contain a multiplicity risk in their primary analysis (66.3% [177 of 267] vs 50.4% [123 of 244]; *P* < .001), and larger trials vs smaller trials were less likely to make any adjustments for multiplicity (35.6% [52 of 146] vs 21.4% [33 of 154]; *P* = .001).

**Conclusions and Relevance:**

Findings from this study suggest that cardiovascular RCTs published in medical journals with high impact factors demonstrate infrequent adjustments to correct for multiple comparisons in the primary end point. These parameters may be improved by more standardized reporting.

## Key Points

**Questions:**

What is the prevalence of multiplicity among cardiovascular randomized clinical trials published in 6 medical journals with a high impact factor, and how frequently are multiplicity adjustments made in these trials?

**Findings:**

In this cross-sectional study, data were collected from the past issues of 6 journals with high impact factors published between August 1, 2015, and July 31, 2018. Of 511 cardiovascular randomized clinical trials included in this analysis, 300 had some form of multiplicity; of these 300, only 85 adjusted for multiplicity.

**Meaning:**

Among contemporary cardiovascular randomized clinical trials, it appears that multiplicity adjustments are infrequently reported.

## Teaser

This cross-sectional study assesses the prevalence of multiplicity and whether appropriate multiplicity adjustments were performed among cardiovascular randomized clinical trials published in 6 medical journals with a high impact factor.

## Introduction

Previous studies^1,2,3,4^ have raised concerns about selective reporting of outcomes in randomized clinical trials (RCTs). However, few reports have focused on multiplicity, which (along with incomplete reporting) is a major factor contributing to nonreproducibility of published claims.^5^ Multiplicity refers to the “potential inflation of type I error rate as a result of multiple testing, for example because of multiple subgroup comparisons, comparisons across multiple treatment arms, analysis of multiple outcomes, and multiple analyses of the same outcome at different times.”^6^

Negative consequences associated with multiplicity could be prevented by complete and accurate reporting of analyses outlined in the registered trial protocols. Multiplicity could also be mitigated by statistical adjustment when multiple analyses are specified a priori. Several statistical methods, such as defining coprimary outcome variables, performing various stepwise procedures,^7,8,9,10^ applying methods for multiple-group comparisons^11,12^ and including gatekeeping or hierarchical testing, have been proposed for multiplicity adjustment.^13,14^

To our knowledge, no study has reported on the prevalence of multiplicity among cardiovascular RCTs and, when applicable, whether appropriate multiplicity adjustments were implemented. To fill this knowledge gap, we conducted a cross-sectional study of cardiovascular RCTs published in medical journals with high impact factors to assess the reporting quality of statistical analyses, including the frequency with which multiplicity adjustments were reported.

## Methods

This cross-sectional study followed the Strengthening the Reporting of Observational Studies in Epidemiology (STROBE) reporting guideline.^15^ It also followed methods from the American Heart Association on standards for cardiac prevention and treatment studies.^16^

### Data Sources and Search Strategy

Three cardiovascular journals (*Circulation*, *European Heart Journal*, and *Journal of the American College of Cardiology*) and 3 general medicine journals (*JAMA*, *The Lancet*, and *The New England Journal of Medicine*) published between August 1, 2015, and July 31, 2018, were searched for general trial characteristics, multiplicity error, and multiplicity correction to assess the pool of recent and contemporary cardiovascular clinical trials. Data were analyzed December 20 to 27, 2018. These journals were chosen based on their high impact factor, broad readership, and reputation of publishing important clinical trials used in the development of guidelines. Supplements and trial protocols of each of the included RCTs were also searched for general trial characteristics, multiplicity error, and multiplicity correction.

### Study Selection

Articles were selected if they reported results of cardiovascular RCTs and compared at least 2 treatment groups. Excluded were brief communications, research letters, and animal studies. Data from the selected RCTs were extracted and verified by 2 of us (M.S.K. and Z.N.A.) independently using a structured data instrument and then cross-checked by another of us (T.J.S).

### Data Extraction

Data were extracted from both the primary and secondary articles. Primary articles were defined as reports on an empirical research study conducted by the authors analyzing data collected for the first time, while secondary articles were studies derived from data collected and analyzed from primary articles. We analyzed and extracted data only from the analysis of the primary end point of each RCT because multiplicity in a secondary analysis is generally exploratory or hypothesis generating. A multiplicity coding manual was developed to investigate the reporting of primary statistical analyses, multiple analyses, and adjustments for multiplicity issues (eAppendix in the Supplement). The multiplicity coding manual was pretested and modified by coding 15 articles initially. Two of us (M.S.K. and Z.N.A.) coded each article separately and discussed any inconsistencies in the data and modified the multiplicity coding manual accordingly. The rest of the articles were then coded according to this multiplicity coding manual. The complete published articles were searched for general trial characteristics, multiplicity error, and multiplicity correction by the coders, along with additional supplementary material (eg, trial protocols and appendixes if they were referred to in the article). The order of the articles was randomized for each coder.

To measure the extent of agreement between the 2 independent coders, the κ statistic was used and calculated according to the methods by Landis and Koch.^17^ The frequency of discrepancies between the coders was computed using the Kappa Calculator (Statistics Solutions),^18^ and the κ statistics were assessed for several outcomes. There was substantial agreement in reproducibility for the presence of multiplicity, with κ = 0.76 (95% CI, 0.51-0.90) in the main text and κ = 0.78 (95% CI, 0.55-0.89) after adjusting for these multiplicity errors. Overall interobserver agreement in extracting data was good, and any discrepancies were resolved after discussion. When consensus could not be reached, another of us (T.J.S.) arbitrated. Finally, a post hoc search was done to assess if the authors of articles stated that their trial was exploratory or hypothesis generating in any section of the article.

### General RCT Characteristics

The following information was extracted from the RCTs: (1) the number of randomized participants; (2) region of the world where the trial was conducted (North America, Western Europe, multiregional, or rest of the world [multiregional was defined as any trial that had multiple sites across the world, and rest of the world was defined as any trial having sites in a region that was not located in either North America or Western Europe]); (3) intervention type (drugs, procedures [eg, a different approach or method of implementing treatment], medical devices, surgery, testing or imaging, or other [eg, diet]); and (4) funding source. Trial size was extracted (as a proxy for trial phase considering its inconsistent definition) and categorized as small (≤500 participants per group) or large (>500 participants per group). Trial type (prespecification of a primary end point) was categorized and extracted as either a mortality trial (defined as any trial where the primary outcome was mortality during treatment) or a nonmortality trial.

### Outcome Data

Data were also extracted for whether the article had risk of multiplicity (ie, contained multiple analyses, a term that encompasses any of the following: multiple treatment groups, multiple outcome variables, and multiple analyses of the same outcome variable) and whether the authors defined the methods used for multiplicity correction. An RCT was considered to have multiple treatment groups if it had more than 2 arms, multiple outcome variables were defined as having more than 1 primary outcome, and multiple analyses were defined as analysis of the same outcome variable in multiple ways. All 3 of these scenarios were weighted equally. We considered multiplicity adjustment sufficient when an article outlined that it attempted to adjust for multiple comparisons.

### Statistical Analysis

Descriptive statistics were used to assess the proportion of RCTs with (1) multiple primary analyses and (2) a multiplicity adjustment for the analysis of the primary end point. Also recorded were the class of multiplicity in the primary analysis (multiple treatment groups, multiple outcome variables, or multiple analyses of the same outcome variable) and frequencies of each of the methods used to adjust for multiplicity in the primary analysis. Multiplicity was examined only for the analysis of the primary end point. It was deemed unnecessary for secondary analyses, which are generally exploratory or hypothesis generating. Outcomes of interest were percentages of primary analyses that performed multiplicity adjustment of primary end points. Two-sided χ^2^ tests were used to examine the association between (1) intervention type, (2) funding source, (3) trial size, and (4) trial type; it was noted whether risk for a familywise error because of multiple comparisons was present and whether the RCT adjusted for multiple comparisons. The method described by Holm^8^ adjusts for multiple comparisons between type of intervention type, funding source, trial size, and trial type. According to this method, the smallest *P* value from all planned comparisons is compared with a significance level of .05 divided by *K*, where *K* represents the number of comparisons to be made. If the null hypothesis is rejected, the next smallest *P* value is compared with a significance level of *P* = .05 divided by *K* minus 1, and so on until the null hypothesis can no longer be rejected. In this scenario, a total of 8 comparisons were made; therefore, the significance level was set to α = .006 (according to .05 ÷ by *K* − 1, where *K* = 8 in this case) in the initial step and to α = .05 in the last step (α being the Holm-corrected significance level). A statistical software package (SPSS, version 23; IBM) was used for all analyses.

## Results

### Literature Search

The initial search identified 2166 trials, which were transferred to a reference management software program (EndNote, Clarivate Analytics). The titles and abstracts of the identified studies were then screened to exclude irrelevant studies. Full-text studies were subsequently obtained and evaluated for the remaining 1273 reports. After assessing for relevance, 511 articles were included in the final analysis.

### Study Characteristics

Of 511 cardiovascular RCTs included in this analysis, 123 (24.1%) were published in *Journal of the American College of Cardiology*, 112 (21.9%) in *Circulation*, 107 (20.9%) in *European Heart Journal*, 71 (13.9%) in *The New England Journal of Medicine*, 55 (10.8%) in *The Lancet*, and 43 (8.4%) in *JAMA* (Table 1). Approximately half (248 [48.5%]) of the trials were industry funded, and approximately half (243 [47.6%]) were large trials. Approximately half (251 [49.1%]) of the trials used a drug intervention. A total of 229 trials (44.8%) made use of composite outcomes as their primary outcome variable.

**Table 1.** Characteristics of 511 Included Randomized Clinical Trials

Variable	No. (%)				
	2015 (n = 106)	2016 (n = 162)	2017 (n = 159)	2018 (n = 84)	Total (N = 511)
Total participants, No.	377 591	596 297	687 921	371 819	NA
Participants per trial, No.	3562	3681	4327	4426	NA
Journals					
Cardiovascular	74/342 (21.6)	105/342 (30.7)	105/342 (30.7)	58/342 (17.0)	342/511 (66.9)
General medicine	32/169 (18.9)	57/169 (33.7)	54/169 (32.0)	26/169 (15.4)	169/511 (33.1)
Region					
North America	9 (8.5)	26 (16.0)	31 (19.5)	14 (16.7)	80 (15.7)
Western Europe	25 (23.6)	39 (24.1)	34 (21.4)	12 (14.3)	110 (21.5)
Multiregional	35 (33.0)	61 (37.7)	57 (35.8)	29 (34.5)	182 (35.6)
Rest of the world	15 (14.2)	11 (6.8)	11 (6.9)	18 (21.4)	55 (10.8)
Not mentioned	22 (20.8)	25 (15.4)	26 (16.4)	11 (13.1)	84 (16.4)
Intervention type					
Drugs	60 (56.6)	79 (48.8)	73 (45.9)	39 (46.4)	251 (49.1)
Procedures	10 (9.4)	23 (14.2)	23 (14.5)	18 (21.4)	74 (14.5)
Medical devices	7 (6.6)	8 (4.9)	8 (5.0)	5 (6.0)	28 (5.5)
Surgery	5 (4.7)	11 (6.8)	7 (4.4)	2 (2.4)	25 (4.9)
Testing or imaging	0	4 (2.5)	7 (4.4)	4 (4.8)	15 (2.9)
Other	24 (22.6)	37 (22.8)	41 (25.8)	16 (19.0)	118 (23.1)
Funding source					
No source	0	1 (0.6)	0	1 (1.2)	2 (0.4)
Government funding	27 (25.5)	52 (32.1)	45 (28.3)	24 (28.6)	148 (29.0)
University or organization	17 (16.0)	25 (15.4)	30 (18.9)	23 (27.4)	95 (18.6)
Industry	58 (54.7)	76 (46.9)	80 (50.3)	34 (40.5)	248 (48.5)
Not mentioned	2 (1.9)	6 (3.7)	1 (0.6)	1 (1.2)	10 (2.0)
Other	2 (1.9)	2 (1.2)	3 (1.9)	1 (1.2)	8 (1.6)
Trial size					
≤500 Participants per group	59 (55.7)	86 (53.1)	80 (50.3)	43 (51.2)	268 (52.4)
>500 Participants per group	47 (44.3)	76 (46.9)	79 (49.7)	41 (48.8)	243 (47.6)

Abbreviation: NA, not applicable.

### Risk of Multiplicity

Of 511 cardiovascular RCTs included in this analysis, 300 (58.7%) had some form of multiplicity (282 of 511 [55.2%] did not mention whether they did or did not adjust for multiplicity). Of these 300 trials, 81 (27.0%) had multiple treatment groups, 45 (15.0%) identified multiple outcome variables as primary, 170 (56.7%) had multiple analyses of the same outcome variable, 3 (1.0%) had multiple treatment groups and multiple outcome variables, and 1 (0.3%) had multiple treatment groups and multiple analyses (Table 2).

**Table 2.** Multiplicity Adjustment

Variable	Frequency, No. (%)
Primary analysis contained multiple analyses, of those that identified a primary analysis (n = 511)	300 (58.7)
Types of multiple analyses included, of those with multiple primary analyses (n = 300)	
Multiple treatment groups	81 (27.0)
Multiple outcome variables	45 (15.0)
Multiple analyses of the same outcome variable	170 (56.7)
Multiple treatment groups and multiple outcome variables	3 (1.0)
Multiple treatment groups and multiple analyses	1 (0.3)
Adjusted for all multiple comparisons, of those with multiple primary analyses (n = 300)	85 (28.3)

### Multiplicity Adjustment

Among 300 RCTs, only 85 (28.3%) adjusted for multiplicity for all primary analyses (Table 2). Of 511 trials, 289 (56.6%) did not mention whether they did or did not attempt to adjust for multiple comparisons. Forty-one trials (48.2%) had multiple analyses of the same outcome variable that adjusted for multiplicity, 22 (25.9%) had multiple treatment groups that adjusted for multiplicity, and 19 (22.4%) had multiple outcome variables that adjusted for multiplicity. The individual multiplicity correction tests are also listed in Table 3.

**Table 3.** Methods Used to Adjust for Multiplicity

Variable	Articles that adjusted for multiplicity, No. (%)					*P* value
	For all primary analyses (N = 85)	With multiple treatment groups (n = 22)	With multiple outcome variables (n = 19)	With multiple analyses of the same outcome variable (n = 41)	With multiple treatment groups and multiple outcome variables (n = 3)	
≥2 Coprimary outcome variables with statistically significant treatment associations	1 (1.2)	0	1 (5.3)	0	0	<.001
Bonferroni adjustment	15 (17.6)	4 (18.2)	2 (10.5)	8 (19.5)	1 (33.3)	<.001
Hochberg test	5 (5.9)	2 (9.1)	1 (5.3)	2 (4.9)	0	<.001
Dunn test	3 (3.5)	2 (9.1)	0	1 (2.4)	0	<.001
Gatekeeping or hierarchical testing	19 (22.4)	5 (22.7)	2 (10.5)	11 (26.8)	1 (33.3)	<.001
Holm test	3 (3.5)	1 (4.5)	1 (5.3)	1 (2.4)	0	.001
Adjusted *P* value to account for multiplicity	26 (30.6)	2 (9.1)	11 (57.9)	13 (31.7)	0	<.001
Coprimary outcome variables and gatekeeping	3 (3.5)	3 (13.6)	0	0	0	<.001
Fixed sequence test	2 (2.4)	0	1 (5.3)	0	1 (33.3)	<.001
Hommel test	2 (2.4)	0	0	2 (4.9)	0	<.001
Dunnett test	3 (3.5)	3 (13.6)	0	0	0	<.001
Tukey test	3 (3.5)	0	0	3 (7.3)	0	<.001

### Limitation Specified of Performing Multiplicity Adjustment

Of 300 trials with multiplicity error risk, 19 (6.3%) were exploratory or hypothesis generating. Twelve of these trials mentioned this exploratory nature in the Discussion section of the article, 5 mentioned it in the Methods section, and 2 mentioned it in more than 1 section of the article. Of the 85 trials that adjusted for multiplicity, 68 (80.0%) mentioned that they adjusted for multiplicity in the main text of the article, and 17 (20.0%) only mentioned it in the supplement or trial protocol.

### Determinants of Performing Multiplicity

Intervention type and funding source had no statistically significant association with the reporting of multiplicity risk adjustment (Table 4). Trials that assessed mortality vs nonmortality outcomes were more likely to contain a multiplicity risk in their primary analysis (66.3% [177 of 267] vs 50.4% [123 of 244]; *P* < .001). Although larger trials had no association with specifying an analysis of the primary end point or containing a multiplicity error risk within their analysis, they were less likely than smaller trials to make any adjustments to correct for multiplicity issues (35.6% [52 of 146] vs 21.4% [33 of 154]; *P* = .001). All of these results were statistically significant after application of the Holm test.

**Table 4.** Comparisons of Reporting for Intervention Type, Funding Source, Trial Size, and Trial Type

Variable	Primary analysis has multiple analyses, No./total No. (%)	Adjusted for multiplicity, No./total No. (%)
Intervention type		
Drugs	157/251 (62.5)	48/157 (30.6)
Procedures	42/74 (56.8)	12/42 (28.6)
Medical devices	17/28 (60.7)	4/17 (23.5)
Surgery	17/25 (68.0)	2/17 (11.8)
Testing or imaging	8/15 (53.3)	1/8 (12.5)
Other	59/118 (50.0)	18/59 (30.5)
*P* value^a^	.27	.22
Funding source		
None	0/2	0/0
Government	88/148 (59.5)	21/88 (23.9)
University or organization	54/95 (56.8)	16/54 (29.6)
Industry	150/248 (60.5)	46/150 (30.7)
Not mentioned	4/10 (40.0)	1/4 (25.0)
Other	4/8 (50.0)	1/4 (25.0)
*P* value^a^	.41	.74
Trial size		
≤500 Participants per group	146/268 (54.5)	52/146 (35.6)
>500 Participants per group	154/243 (63.4)	33/154 (21.4)
*P* value^a^	.04	.001
Trial type		
Mortality	177/267 (66.3)	47/177 (26.6)
Nonmortality	123/244 (50.4)	38/123 (30.9)
*P* value^a^	<.001	.09

^a^ *P* values from χ^2^ tests. For 8 comparisons, the ordered *P* values are compared with the Holm-corrected significance levels set at the following values until a comparison fails to reach significance: .0062, .007, .008, .01, .0125, .016, .025, and .05.

## Discussion

Our report demonstrates that 58.7% of 511 cardiovascular RCTs included in this analysis contained multiple analyses within their methods and that 55.2% of the total RCTs did not report whether they adjusted for multiple comparisons. Trials that assessed mortality were more likely than nonmortality trials to have some form of multiplicity, which is not unexpected because mortality is usually not the sole end point. However, because of the exigent nature of the mortality component and because some researchers consider mortality a safety end point as well, authors might be inclined to claim an association even if the overall end point fails to show effectiveness. These results have important implications for the performance and interpretation of cardiovascular RCTs.

Articles mentioning that they did not adjust for multiplicity often provided some justification, such as stating that their study was exploratory or hypothesis generating. Some justifications were unique to the trial; for example, 1 article mentioned that a chance finding could not be ruled out because of multiple testing and the sample size of the subgroups.^19^ It is possible that results might change before and after multiplicity adjustment; for example, in a trial where *P* = .046 for the primary outcome, the adjusted *P* value may have been different after multiplicity correction.^20^

Among 85 of 511 included articles that adjusted for multiplicity for all primary analyses (Table 3), half of the trials used a composite outcome as their primary outcome. Composite outcomes allow increased statistical precision and efficiency with fewer participants to detect a statistically significant difference among comparators,^21^ especially in the case of total mortality, which is a rare event requiring more power and an extended follow-up to show a difference between interventions.^22^ Although the use of composite end points is acceptable and in some instances beneficial, it may also increase the risk of introducing a multiplicity error if the observed treatment effect was associated with a softer clinical end point.^21,23^ For example, a trial where all-cause mortality, myocardial infarction, and recurrent angina are components of a composite end point, recurrent angina might be considered the softest of the 3 components. The present study did not examine the details of each composite end point of every trial or consider composite end points to be a source of multiplicity; however it suggests the need for future investigators and researchers to apply methods to avoid the possibility of a multiplicity error, as described by Sankoh et al.^21^

Uncertainty in interpreting research results is common and may be attributable to a lack of statistical power or the use of questionable research practices, or it may reflect decisions a researcher makes to conduct a trial.^24^ These uncertainties might explain gaps in the reporting of multiplicity and adjustments made. Conversely, one could also argue that such gaps are less a reflection of multiplicity issues but rather reflect the unavailability of the trial protocol and statistical analysis plan. We suggest that all RCTs in medical journals should describe the trial protocol–specific analytic plan, including the methods used to adjust for multiple comparisons or acknowledgment of the lack of correction for multiplicity. We believe that this inclusion is especially relevant because most clinicians do not have statistical expertise.

Because the criteria used to classify trial phase (ie, phase 1, 2, or 3) were inconsistent among the RCTs in this study, trial size was used as a proxy for trial phase. For drug interventions, smaller trials (≤500 participants per group) may more likely reflect early to middle stages of development, and larger trials (>500 participants per group) may more likely reflect confirmatory stages.^25,26^ Among 161 RCTs, Gewandter et al^27^ found no association between trial size and funding source, with multiplicity adjustment most likely because of the limited power of a study to perform such an analysis. Our analysis included a larger sample of both industry-sponsored RCTs (n = 248) and large RCTs with multiplicity issues (n = 154) (Table 4). We found that smaller trials were more likely to be adjusted for multiplicity. Funding source had no association with adjusting for multiplicity. This observation suggests that RCTs of drugs in early to middle stages of development may be more likely to adjust for multiplicity.

The appropriateness of testing procedures is guided by information on statistical features of a study design or analytic strategy and differs depending on whether there is a single source of multiplicity or several sources and whether there are multiple treatment groups, multiple outcome variables, or multiple analyses of the same outcome variable. Dmitrienko and D’Agostino^23^ provide some guidance on how to choose the most appropriate test for multiplicity corrections; they state that nonparametric tests, such as the Holm test, can be applied to most multiplicity problems involving a single source of multiplicity. In cases where the association between statistical tests is known, such as in clinical trials with several dose-control comparisons and patient populations, more specific parametric tests, such as the Dunnett test, may be applied. In an effort to better explain types of multiple analyses and multiple outcome variables, detailed examples are listed in eTable 1 in the Supplement.

### Limitations

This study has several limitations. First, we assessed the reporting quality of the methods used for multiplicity adjustments but not necessarily the quality of statistical practices used. Because the methods may have been prespecified but not stated in the articles,^28,29^ this report is subject to reporting bias. Second, studies were recorded as having adjusted for multiplicity in the primary analysis only if the authors adjusted for all instances of multiplicity. However, this approach does not consider the trials that tried to adjust for some but not all sources of multiple comparisons. Also, whether a study had multiple treatment groups, multiple outcome variables, or multiple analyses of the same outcome variable, all had the same weight in terms of adjusting for multiplicity and thus were considered equally. A third limitation is that we only evaluated the primary outcomes. It is important to note that the secondary end point in a sequence may often influence a conclusion. For instance, if a trial finds a statistically significant difference in major adverse cardiovascular events (myocardial infarction, stroke, and admission for heart failure) and the next end point in the sequence is the myocardial infarction rate, which is not statistically significant, it would be incorrect to conclude a nominally statistically significant stroke association. In addition, we were unable to differentiate our analysis by trial type. Although the objective of this study was to evaluate the overall prevalence of multiplicity among cardiovascular RCTs, it must be remembered that phase 3 trials hold the most importance from a public health perspective, and multiplicity is of lesser concern in phase 2 trials.

## Conclusions

This cross-sectional study found frequent inconsistencies associated with multiplicity in primary analysis reporting among cardiovascular RCTs published in medical journals with a high impact factor. These findings adversely reflect on the robustness of data published in journals that carry global reach and generate evidence that can transform clinical guidelines and practice. Our findings suggest that investigators should be encouraged to adjust for multiplicity when warranted. Practical guidelines for multiplicity adjustment in clinical trials (eg, recommendations by Proschan and Waclawiw^30^) can be consulted. We think that this information should ideally be prespecified in the Methods section of clinical trials before unblinding of the study data (eTable 2 in the Supplement). We believe that it should be the collective responsibility of journal editors, peer reviewers, and readers to pay close attention to the Methods and Statistical Analysis sections of articles reporting clinical trial results to ensure that multiplicity issues have been addressed.

## Notes

Supplement. **eAppendix.** Multiplicity Coding Manual **eTable 1.** Examples of Multiple Analyses and Multiple Outcome Variables **eTable 2.** Recommendations on Multiplicity Error in Clinical Trials **eReferences.**
