# Supplementary material for: Prevalence of Multiplicity and Appropriate Adjustments Among Cardiovascular Randomized Clinical Trials Published in Major Medical Journals
Source: JAMA Netw Open. 2020 Apr 17;3(4):e203082. doi: 10.1001/jamanetworkopen.2020.3082 (PMC7165301; doi:10.1001/jamanetworkopen.2020.3082)
Supplement: Supplement. — eAppendix. Multiplicity Coding Manual eTable 1. Examples of Multiple Analyses and Multiple Outcome Variables eTable 2. Recommendations on Multiplicity Error in Clinical Trials eReferences. [file jamanetwopen-3-e203082-s001.pdf]

# Supplementary Online Content

Khan MS, Khan MS, Ansari ZN, et al. Prevalence of multiplicity and appropriate adjustments among cardiovascular randomized clinical trials published in major medical journals. *JAMA Netw Open*. 2020:3(4):e203082. doi:10.1001/jamanetworkopen.2020.3082

**eAppendix.** Multiplicity Coding Manual

**eTable 1.** Examples of Multiple Analyses and Multiple Outcome Variables

**eTable 2.** Recommendations on Multiplicity Error in Clinical Trials

**eReferences.**

This supplementary material has been provided by the authors to give readers additional information about their work.

© 2020 Khan MS et al. *JAMA Network Open*.

**eAppendix.** Multiplicity Coding Manual

1 – Study name

2- Year

3- Citations

4- Pubmed indexed

1 = Yes

2 = No

5- Total participants

6-Journal

1 = NEJM

2 = Circulation

3 = Lancet

4 = JACC

5 = JAMA

6 = Eur Heart Journal

7- Number of arms

8- Intervention

Whether the study is comparing drugs, procedures, medical devices, or awareness interventions

1 = Drugs

2 = Procedures

3 = Medical devices

4 = Surgery

5 = Testing/Imaging

© 2020 Khan MS et al. *JAMA Network Open*.

6 = Other

9 – Type of blinding

Whether the study uses single (patient doesn’t know which group he is in), double (patient and doctor both don’t know) or triple blinding (patient, doctor and analysis team doesn’t know)

0 = No blinding

1 = Single blinding

2 = Double blinding

3 = Not mentioned

10 – Region

1 = RestOfWorld

2 = Multiregional

3 = NorthAmerica

4 = WesternEurope

11 – ITT or PTT

1 = ITT

2 = PTT

3 = Both

4 = Not mentioned

12 – Enrollment rate given

1 = Yes

2 = No

13 – Funding

1 = No source

© 2020 Khan MS et al. *JAMA Network Open*.

2 = Government funding

3 = University/Organization

4 = Industry

5 = Not mentioned

6 = Other

14 – Primary outcome

1 = Mortality

2 = Non-mortality/Intermediate

3 = Surrogate

4 = Not mentioned

15 – Composite outcome

1 = Yes

2 = No

16 – Specified primary analysis

1 = Yes

2 = No

17 – Primary analysis has multiple analysis

1 – Yes

2 – No

18 – Adjusted for multiple comparisions

1 = Yes

2 = No

3 = Not mentioned

19- Type of multiple analysis

© 2020 Khan MS et al. *JAMA Network Open*.

1 = Multiple groups

2 = Multiple outcomes

3 = Multiple analysis of the same outcome variable

0 = None

A coding manual was developed to investigate the reporting of primary statistical analysis, multiple analysis and adjustments for multiplicity issues. When a primary analysis was identified, questions regarding multiplicity were answered only in reference to the stated primary analysis. The coding manual was pretested and modified by coding 15 articles initially. Two researchers (K.M.S and A.Z.N) coded each article separately and discussed any inconsistencies in the data and modified the manual accordingly (Appendix).

© 2020 Khan MS et al. *JAMA Network Open*.

**eTable 1.** Examples of Multiple Analyses and Multiple Outcome Variables

| **Multiple analyses** | **Multiple outcomes variables** |
|---|---|
| Ellis et al. [1], in order to adjust for multiplicity, they considered the trial as two independent studies. 1) A clinical study based on the primary analysis group of the ABSORB III (~2000 subjects) and 2) an imaging study based on the pooled population the imaging cohort of the ABSORB III (~200 subjects, which is separate from the ~2000 subjects of the primary analysis group) and the ABSORB japan RCT (~400 subjects). | Zhu et al. [6] assessed three primary outcome variables; change in lesion volume at day 1, hemorrhage volume at day 1 and NIHSS (National institutes of health stroke scale) change at day 1. |
| The apixaban for reduction in stroke and other thromboembolic events in atrial fibrillation (ARISTOTLE) trial was a randomized control trial with patients receiving either warafin or apixaban. One study did a secondary analysis of data from the ARISTOTLE trial to assess how the differences between sexes might have influenced results. [2] | Valgimigl et al. [7] measured two primary endpoints; MACE and net adverse clinical events (a composite of major bleeding or a major adverse cardiovascular event). |
| Hess et al's [3] study was a post-hoc analysis assessing whether the effect of apixaban on efficacy and safety outcomes varied by the concomitant antiplatelet regimen, which was assessed using simple cox modeling and | Basaria et al. [8] assessed co-primary outcomes; common carotid artery intima-media thickness and coronary artery calcium. |

© 2020 Khan MS et al. *JAMA Network Open*.

| | |
|---|---|
| marginal structural models with propensity scores with antiplatelet therapy as a time-dependent covariate. | |
| In the PARADIGM-HF ,8399 patients in new york heart association functional class II–IV with an LVEF ≤40% were randomized to either enalapril or LCZ696, an analysis was then conducted which examined the pre-specified efficacy and safety outcomes according to age category: <55, 55–64, 65–74, and ≥75. [4] | Cowie et al [9] evaluated three primary outcomes; the time to first event of: 1) all cause mortality or unplanned hospitalization for worsening heart failure 2) cardiovascular mortality or unplanned hospitalization for worsening heart failure 3) all cause mortality or all cause hospitalization |
| Peñalvo JL et al [5] evaluated changes after the intervention (SI! Program intervention) on children, analyses were conducted in patients who had data for the primary outcome at baseline and after 1, 2, and 3 years. | Michael et al. [10] measured co-primary end points; rate of hepatotoxicity and renal toxicity. |

PARADIGM-HF: Prospective comparison of angiotensin receptor neprilysin inhibitor (ARNI) with angiotensin converting enzyme inhibitor to determine impact on global mortality and morbidity in heart failure trial

MACE: Major adverse cardiovascular events (a composite of death, myocardial infarction, or stroke)

LVEF: Left ventricular ejection fraction

© 2020 Khan MS et al. *JAMA Network Open*.

**eTable 2.** Recommendations on Multiplicity Error in Clinical Trials

| **Recommendations** | |
|---|---|
| **Investigators and authors** | Pre-specify analyses |
| | Pre-specify methods used for multiplicity adjustment, when applicable |
| | Clearly report the above methods in the methods section of publications and indicate whether they were pre-specified |
| | Acknowledge in a publication use of a composite end point may lead to misinterpretation (e.g. assumption that each component of the composite endpoint is equally affected) |
| **Reviewer** | Pay close attention to the methods and limitations section of submitted manuscripts and require authors to follow the above recommendations |
| **Editors** | Require the above recommendations for publications and enforce these requirements |
| | Consider requiring that authors submit a copy of the trial protocol along with the manuscript to ensure consistency in statistical methods used |

© 2020 Khan MS et al. *JAMA Network Open*.

**eReferences**


1. Ellis SG, Kereiakes DJ, Metzger DC, et al. Everolimus-eluting bioresorbable scaffolds for coronary artery disease. *New England Journal of Medicine.* 2015;373(20):1905-1915.
2. Vinereanu D, Stevens SR, Alexander JH, et al. Clinical outcomes in patients with atrial fibrillation according to sex during anticoagulation with apixaban or warfarin: a secondary analysis of a randomized controlled trial. *European heart journal.*2015;36(46):3268-3275.
3. Hess CN, James S, Lopes RD, et al. Apixaban plus mono versus dual antiplatelet therapy in acute coronary syndromes: insights from the APPRAISE-2 trial. *Journal of the American College of Cardiology.*2015;66(7):777-787.
4. Jhund PS, Fu M, Bayram E, et al. Efficacy and safety of LCZ696 (sacubitril-valsartan) according to age: insights from PARADIGM-HF. *European heart journal.* 2015;36(38):2576-2584.
5. Peñalvo JL, Santos-Beneit G, Sotos-Prieto M, et al. The SI! Program for cardiovascular health promotion in early childhood: a cluster-randomized trial. *Journal of the American College of Cardiology.*2015;66(14):1525-1534.
6. Zhu Z, Fu Y, Tian D, et al. Combination of the immune modulator fingolimod with alteplase in acute ischemic stroke: a pilot trial. *Circulation.*2015;132(12):1104-1112.
7. Valgimigli M, Frigoli E, Leonardi S, et al. Bivalirudin or unfractionated heparin in acute coronary syndromes. *New England Journal of Medicine.*2015;373(11):997-1009.
8. Basaria S, Harman SM, Travison TG, et al. Effects of testosterone administration for 3 years on subclinical atherosclerosis progression in older men with low or low-normal testosterone levels: a randomized clinical trial. *Jama.*2015;314(6):570-581.
9. Cowie MR, Woehrle H, Wegscheider K, et al. Adaptive servo-ventilation for central sleep apnea in systolic heart failure. *New England Journal of Medicine.*2015;373(12):1095-1105.


© 2020 Khan MS et al. *JAMA Network Open*.

10. Michael Gibson C, Korjian S, Tricoci P, et al. Safety and tolerability of CSL112, a reconstituted, infusible, plasma-derived apolipoprotein AI, after acute myocardial infarction: the AEGIS-I trial (ApoA-I Event Reducing in Ischemic Syndromes I). *Circulation.*2016;134(24):1918-1930.

© 2020 Khan MS et al. *JAMA Network Open*.
